# EBV-Derived miR-BART20-3p Influences Proliferation and Migration in EBV-Positive Gastric Cancer Models by Suppressing PPARα

**DOI:** 10.3390/microorganisms13071514

**Published:** 2025-06-28

**Authors:** Qiong Wu, Guiying Ye, Xiazhen Xu, Xianchang Zeng, Biyun Wu, Fan Xin, Lu Zhang, Xu Lin, Xinjian Lin, Wannan Chen

**Affiliations:** 1 Key Laboratory of Gastrointestinal Cancer (Fujian Medical University), Ministry of Education, School of Basic Medical Sciences, Fujian Medical University, 1 Xue Fu North Road, Fuzhou 350122, China; joan_w2021@fjmu.edu.cn (Q.W.); 13039242025@fjmu.edu.cn (G.Y.); xuxiazhen123@fjmu.edu.cn (X.X.); zengxianchang.ykdx@fjmu.edu.cn (X.Z.); wubiyun@stu.fjmu.edu.cn (B.W.); xinfan@fjmu.edu.cn (F.X.); canarymf28@163.com (L.Z.); linxu@mail.fjmu.edu.cn (X.L.); 2 Fujian Key Laboratory of Tumor Microbiology, Department of Medical Microbiology, School of Basic Medical Sciences, Fujian Medical University, 1 Xue Fu North Road, Fuzhou 350122, China

**Keywords:** Epstein–Barr virus-encoded miRNAs, miRNA-BART20-3p, gastric cancer, PPARα, IL-6, cell proliferation, migration

## Abstract

Epstein–Barr virus (EBV) is the first oncogenic DNA virus known to encode microRNAs (miRNAs) and has been implicated in the pathogenesis of multiple malignancies, including a distinct subset of gastric cancers (EBV-associated gastric cancer, EBVaGC). However, the functional roles of individual EBV-encoded miRNAs in EBVaGC remain poorly defined. In this study, we integrate bioinformatic and experimental analyses to uncover a novel oncogenic axis driven by EBV-encoded miR-BART20-3p. Analysis of public transcriptomic datasets revealed that peroxisome proliferator-activated receptor α (PPARα) is significantly downregulated in EBVaGC compared with EBV-negative gastric tumors. We confirmed that both PPARα mRNA and protein are reduced in EBVaGC cell lines and primary tumor specimens, and that this reduction inversely correlates with miR-BART20-3p levels. A dual-luciferase reporter assay demonstrated that miR-BART20-3p directly binds the PPARα 3′-UTR. Functionally, miR-BART20-3p overexpression in AGS cells enhanced proliferation and migration, whereas inhibition of miR-BART20-3p in EBV-infected AGS cells attenuated these phenotypes. Mechanistic studies employing PPARα-specific siRNA together with qRT-PCR and ELISA reveal that suppression of PPARα or overexpression of miR-BART20-3p leads to upregulation of interleukin 6 (IL-6), indicating disruption of the PPARα–IL-6 regulatory axis. Collectively, EBV-encoded miR-BART20-3p promotes EBVaGC progression by directly targeting PPARα, and thereby derepressing IL-6 expression. This miRNA–PPARα–IL-6 pathway may serve as both a mechanistic biomarker and a novel therapeutic target in EBVaGC.

## 1. Introduction

Gastric cancer (GC) ranks fifth worldwide in both incidence and mortality, with particularly high rates in East Asia [1]. Its etiology is multifactorial, encompassing environmental factors, dietary habits, and infectious agents such as Helicobacter pylori and Epstein–Barr virus (EBV) [2]. In 2014, The Cancer Genome Atlas (TCGA) subdivided GC into four molecular subtypes: EBV-positive, microsatellite instability–high (MSI-H), genomically stable (GS), and chromosomal instability (CIN) [3]. Approximately 9% of GC cases are EBV-associated (EBVaGC), which exhibit unique pathological and molecular features driven by clonal proliferation of EBV-infected cells [3,4]. Despite this classification, the mechanisms by which EBV infection promotes gastric tumorigenesis remain incompletely understood, prompting recent efforts to elucidate EBVaGC pathogenesis for improved diagnosis, treatment, and prevention.

EBV is a ubiquitous γ-herpesvirus transmitted primarily via saliva; over 90% of adults harbor a lifelong latent infection [5,6,7]. Latency programs (types 0–III) differ by expression of EBV nuclear antigens (EBNAs), latent membrane proteins (LMPs), EBV-encoded small RNAs (EBERs), and BamHI A rightward transcripts (BARTs), including BART microRNAs (miRNAs) [8]. In EBVaGC specimens, the virus adopts an intermediate latency state expressing EBNA1, LMP1, LMP2A/B, and abundant BART miRNAs [9], which sustain latency, evade immune surveillance, and influence host cell growth and invasion.

MiRNAs are ~22-nucleotide noncoding RNAs that bind target mRNA 3′-untranslated regions to trigger degradation or translational repression [10]. Dysregulated miRNAs contribute to oncogenesis and may serve as biomarkers or therapeutic targets. EBV was the first virus shown to encode miRNAs, with 25 precursor and 44 mature miRNAs clustered in the BHRF1 and BART regions [11]. Elevated BART miRNA expression in EBVaGC underscores their oncogenic potential [12], yet the specific functions and regulatory networks of individual BART miRNAs in EBVaGC require further investigation.

Peroxisome proliferator-activated receptor α (PPARα) is a nuclear receptor that governs fatty acid β-oxidation and lipoprotein metabolism in liver, kidney, and heart [13,14]. Beyond metabolic regulation, PPARα exerts anti-inflammatory effects by repressing cytokines such as TNF-α, IL-1β, and IL-6 [15,16]. Clinical studies report reduced circulating PPARα levels in GC patients, and epidemiological data link aspirin’s enhancement of PPARα activity to decreased GC risk [17,18]. These observations suggest that diminished PPARα expression may facilitate inflammation-driven gastric tumorigenesis.

In the present study, we performed integrated bioinformatic analyses of TCGA and GEO datasets to identify host genes downregulated in EBVaGC versus EBV-negative GC (EBVnGC). Candidate genes were cross-referenced with predicted EBV miRNA targets in ViRBase, highlighting PPARα as a putative target of miR-BART20-3p. We then validated that miR-BART20-3p binds the PPARα 3′-UTR to suppress its expression and demonstrated that miR-BART20-3p promotes proliferation, migration, and IL-6 upregulation via PPARα inhibition in gastric cancer cells. These findings uncover a miR-BART20-3p–PPARα–IL-6 axis driving EBVaGC progression and suggest novel avenues for targeted therapy and biomarker development.

## 2. Materials and Methods

### 2.1. Cell Culture and Reagents

AGS and HGC27 are EBV-negative gastric cancer cell lines were purchased from American Type Culture Collection (Manassas, VA, USA). SNU719 is gastric cancer cell line naturally infected with EBV was purchased from FuHeng biology (Shanghai, China). AGS-EBV cells were established through infection of AGS cells with recombinant EBV derived from the Burkitt’s lymphoma cell line Akata, kindly provided by professor Zeng Musheng. AGS-EBV cells were maintained in DMEM/F12 medium with 400 µg/mL G418 (Gibco, Carlsbad, CA, USA) to sustain EBV expression. AGS and HGC27 cells were maintained in DMEM/F12 medium, SNU719 cells were cultured in RPMI-1640 medium. Each cell line was cultured under standard conditions using the recommended growth medium containing 10% fetal bovine serum (PAN, Aidenbach, Germany). BART20-3p mimic and inhibitor were purchased from GenePharma (Shanghai, China).

### 2.2. Construction of AGS-EBV Cell Line

Dishes of 35 mm were used to seed AGS cells at a density of 2 × 10^5^ cells per dish, which were then cultivated until they reached roughly 70% confluence. Subsequently, Akata cells were used to produce high green fluorescence of recombinant EBV virions pretreated with 0.5% anti-human IgG (Yeasen, Shanghai, China) for 24 h, then were introduced into the AGS cultures and co-cultured for 2–3 days. The residual Akata cells were then eliminated through repeated washing with 1× PBS, followed by continuous selection with 400 μg/mL G418 for two weeks.

### 2.3. In Situ Hybridization for EBER

AGS and AGS-EBV cells were collected and fixed overnight in 3.7% neutral formaldehyde. Following PBS washes, the cell pellets were resuspended in 2.5% agarose and placed into tissue embedding cassettes. The resulting blocks were sectioned into 3–4 μm slices using a microtome. Paraffin sections were deparaffinized in absolute ethanol, air-dried, and then blocked in the dark for 10 min. Following graded ethanol dehydration, the sections were incubated with pepsin working solution at 37 °C for 15 min. After a second dehydration and air-drying, digoxigenin-labeled EBER probe kits (ZSGB-BIO, Beijing, China) were applied for hybridization at 37 °C for 2–4 h. After washing with 1× PBS, HRP-conjugated anti-digoxigenin antibodies were added and incubated at 37 °C for 30 min. Sections were then washed with PBS, followed by development using freshly prepared DAB substrate. Signal detection was performed under a light microscope.

### 2.4. Immunohistochemistry (IHC)

Clinical gastric cancer tissue specimens used in this study were obtained from the First Affiliated Hospital of Fujian Medical University, with approval from the institutional ethics committee. The collected samples were paraffin-embedded and serially sectioned at a thickness of 3–4 μm using a microtome. Sections were mounted onto glass slides and subjected to antigen retrieval in pH 9.0 EDTA buffer (ZSGB-BIO), using high-pressure steam sterilization for 3 min. Blocking solution was applied and incubated at 37 °C for 20 min, then incubated overnight at 4 °C with a primary antibody against PPARα (Abcam, Cambridge, UK). The next day, slides were washed with PBS and incubated with an HRP-conjugated secondary antibody (ZSGB-BIO) for 20 min. Color development was achieved using freshly prepared DAB substrate kit. After rinsing with distilled water, the nuclei were counterstained with hematoxylin. Sections were then dehydrated through a graded ethanol series, and sealed with neutral mounting medium. Stained sections were observed and imaged under a light microscope for analysis.

### 2.5. Bioinformatics Analysis of DEGs Between EBVnGC and EBVaGC

To identify DEGs between EBVnGC and EBVaGC, transcriptomic data were obtained from the TCGA database, including 224 EBVnGC and 23 EBVaGC tissue samples, classified based on EBV infection status (referred to as the TCGA-EBV dataset). Additionally, datasets GSE51575 comprising 14 EBVnGC and 12 EBVaGC tissue samples, and were retrieved from the GEO database for further analysis. The gene expression profile matrix files from the TCGA-EBV dataset and GSE51575 were processed using Perl. Through Perl (v5.41.3) scripts and corresponding gene annotation, gene probe IDs were converted to gene symbols across these datasets. The “limma (v3.58.1)” and “edgeR (v4.6.2)” packages in R (v4.3.3) were utilized to identify DEGs between EBVnGC and EBVaGC samples in the TCGA-EBV and GSE51575 datasets. Data normalization was conducted using the Robust Multi-Array Average (RMA) method.

To minimize false positives, the Benjamini–Hochberg correction was used to modify the significance *p*-values from hypothesis testing. DEGs were identified based on the False Discovery Rate (FDR) and Fold Change (FC) criteria, which quantify expression differences between sample groups. Genes meeting the thresholds of FDR < 0.05 and |log2(FC)| ≥ 1 were classified as DEGs. To enhance the robustness of the identified DEGs between EBVnGC and EBVaGC, the “VennDiagram (v1.7.3)” package was employed to determine overlapping DEGs across the TCGA-EBV and GSE51575 datasets, with the genes that overlap being chosen for further investigation.

### 2.6. Analysis of EBV-Encoded miRNAs Mediated Regulatory Network

The ViRBase database was accessed and utilized to identify potential host gene regulatory targets of EBV-encoded miRNAs. Subsequently, these target genes were cross-analyzed with DEGs downregulated in EBVaGC relative to EBVnGC to confirm those downregulated DEGs regulated by EBV-encoded miRNAs. Finally, the resulting regulatory network was visualized and presented using the MCODE plug-in (version 1.5.1) within Cytoscape (version 3.7.1, https://cytoscape.org/).

The GSE62254 expression data was downloaded from the GEO database and preprocessed using the limma R installation package to extract specific gene expression profiles of 257 EBVnGC and 18 EBVaGC tissue samples. In the GSE62254 dataset, the expression of previously identified downregulated DEGs mediated by EBV miRNAs was evaluated. The Wilcoxon rank sum test (Mann–Whitney U test) was used to compare the miRNA-associated down-regulation of DEGs expression levels, and the results were visualized using box plots.

### 2.7. Target Prediction

The PPARα sequence (NM_005036.6) used for miRNA target prediction was sourced from the National Center for Biotechnology Information database. The minimum free energy of hybridization needed for miRNAs to bind to particular RNAs was determined using a publicly available RNA hybridization tool (http://bibiserv.techfak.uni-bielefeld.de/rnahybrid/, accessed on 25 August 2024). This tool was then employed to investigate the sequence of the 3′-UTR of PPARα targeted by EBV-encoded miRNAs.

### 2.8. Luciferase Reporter Assay

AGS or AGS-EBV cells were co-transfected with either 20 nM of BART20-3p mimic or 20 nM BART20-3p inhibitor, along with luciferase reporter constructs pGL4.10 vector (Promega, Madison, WI, USA) inserted with wild-type or mutant PPARα. At 48 h post-transfection, luciferase activity was measured using the Dual-Glo luciferase reporter assay system (Promega). For each sample, Renilla luciferase activity was adjusted to firefly luciferase activity. The sequences of the miR-BART20-3p mimic, inhibitor, and their corresponding negative controls used in this study are listed in Table S1.

### 2.9. Quantitative Reverse Transcription PCR (qRT-PCR)

Following the manufacturer’s protocol, total RNA was extracted from cells and tissues using RNA reagent (Invitrogen, Waltham, MA, USA). Quantitative reverse transcription PCR (qRT-PCR) was conducted using a SYBR Green PCR kit (Agbio, Hunan, China). Relative gene expression levels were estimated based on quantification cycle (Cq) values. Then, the reverse transcription primers for miR-BART20-3p were 5′-GTCGTATCCAGTGCAGGGTCCGAGGTATTCGCAATGGATACGACGGTAAC-3′, and the reference miRNA U6 reverse transcription primers were 5′-GTCGTATCCAGTGCAGGGTCCGAGGTATTCGCACTGGATACGACAACGCT-3′. Primer sequences employed in the real-time PCR analysis are presented in Table S2.

### 2.10. ELISA

The levels of IL-6 in cell culture supernatants were measured using an IL-6 ELISA kit (Spbio, Wuhan, China). A 10 μL aliquot of supernatant was diluted to a final volume of 50 μL and added to the ELISA microplate. After washing the wells with the provided buffer, 50 μL of enzyme conjugate was added to each well, followed by incubation at 37 °C for 30 min. Wells were washed repeatedly to remove residual liquid. Subsequently, a color-developing reagent was added, and the plate was incubated at 37 °C for 10 min in the dark to allow color development. The reaction was terminated, and absorbance was measured at 450 nm using a multimode plate reader (Perkinelmer, Springfield, IL, USA). A standard curve was generated based on the absorbance of the standards, and IL-6 concentrations in the test samples were calculated accordingly.

### 2.11. RNA Interference

Two small interfering RNAs (siRNAs) targeting PPARα (siPPARα#1 and siPPARα#2) were synthesized by GenePharma (Shanghai, China). AGS cells underwent transfection with 30 nM siRNA utilizing INTERFER transfection reagent (Polyplus, Shanghai, China) according to the manufacturer’s instructions. Cells were harvested 48 h after transfection to assess PPARα mRNA and protein expression. Nonspecific siRNA (siNC) was utilized as negative control. The sequences of siRNAs are listed in Table S2.

### 2.12. CCK8 Assay

A total of 1 × 10^3^ cells were seeded into 96-well plates and transfected with either 20 nM BART20-3p mimic or 20 nM BART20-3p inhibitor, along with the corresponding control. After 48 h, the culture medium was discarded, and 10 μL of CCK-8 reagent (Dojindo, Tokyo, Japan) diluted with 90 μL of culture medium was added per well. The cells were maintained in a humidified dark environment at 37 °C for 2 h. The absorbance at 450 nm was subsequently measured using a multimode plate reader (Perkinelmer).

### 2.13. Wound Healing Assay

Cells were seeded into 35 mm plates and transfected with either 20 nM BART20-3p mimic or BART20-3p inhibitor, as well as relative controls. After 6 h, a germ-free 200 µL pipette tip was employed to introduce a scratch in the confluent monolayer, followed by washing with phosphate-buffered saline to remove cell debris. The cells were then cultured under normal culture conditions. The wounds were observed immediately (0 h), as well as at 12 h and 36 h post-scratch. Photographs were captured to assess cell migration across transfection, and wound lengths were measured using ImageJ software version 1.52p (NIH, Bethesda, MD, USA).

### 2.14. Transwell Assay

Cell migration was conducted using Transwell plates (BD Biosciences, Franklin Lakes, NJ, USA). Following transfection with 20 nM miR-BART20-3p mimic or inhibitor, AGS or AGS-EBV cells were resuspended in 400 μL of serum-free DMEM/F12 medium and seeded into the upper chamber. The bottom chamber was filled with 600 μL of DMEM/F12 containing 10% fetal bovine serum (FBS) as a chemoattractant. After 2 h of incubation, non-migrated cells remaining on the upper side of the membrane were gently removed with a cotton swab. Migrated cells on the underside of the chambers were fixed with 4% paraformaldehyde for 30 min and subsequently stained with 0.1% crystal violet for 20 min. The number of migrated cells was quantified under a light microscope (Nikon, Tokyo, Japan) at the appropriate magnification.

### 2.15. Statistical Analyses

The data were analyzed using Student’s *t*-test, and a two-way analysis of variance (ANOVA) was used for the CCK8 assay. Statistical analyses were processed with GraphPad Prism version 7 (GraphPad Software, La Jolla, CA, USA), considering *p*-values < 0.05 as statistically significant. All results were presented as the mean ± standard deviation (SD).

## 3. Results

### 3.1. Bioinformatic Identification of Host Genes Downregulated in EBVaGC and Putatively Targeted by EBV miRNAs

**Figure 1:**
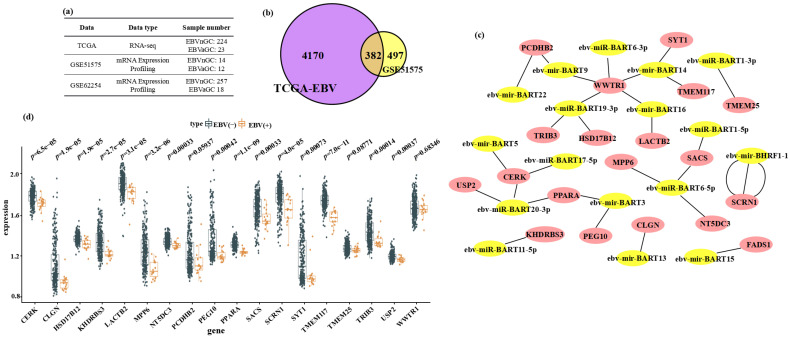
Analysis of downregulated DEGs associated with EBV miRNAs in gastric cancer. (**a**) Overview of sample composition for EBV-negative GC (EBVnGC) and EBV-associated GC (EBVaGC) datasets from TCGA, GSE51575, and GSE62254. (**b**) Venn diagram showing intersection of downregulated genes identified by limma/edgeR analysis in TCGA-EBV and GSE51575 cohorts, yielding 382 shared DEGs. (**c**) Protein–protein interaction network of 382 downregulated DEGs overlaid with predicted EBV-encoded miRNA targets from ViRBase, highlighting 19 genes linked to 17 viral miRNAs. (**d**) Boxplots comparing expression of 18 candidate DEGs in EBVnGC versus EBVaGC samples from GSE62254.

We first retrieved RNA-seq profiles from TCGA (224 EBV–negative GC and 23 EBV-positive GC samples) and GEO dataset GSE51575 (14 EBV–negative and 12 EBV-positive samples) (Figure 1a). Differential expression analysis using the limma and edgeR packages identified 794 upregulated and 4552 downregulated genes in TCGA, and 1088 upregulated and 879 downregulated genes in GSE51575. To pinpoint host genes consistently repressed in EBV-associated GC, we took the intersection of downregulated genes from both cohorts, yielding 382 shared candidates (Figure 1b). We then cross-referenced these 382 genes against the full set of predicted targets for all 44 EBV-encoded miRNAs in the ViRBase database, which catalogs viral miRNA–host mRNA interactions. This step resulted in 19 downregulated genes potentially regulated by 17 EBV miRNAs (8 mature and 9 precursor miRNAs) (Figure 1c). Next, we used an independent cohort (GSE62254; 257 EBV–negative and 18 EBV-positive GC samples) to validate these 19 candidates. Eighteen genes remained downregulated in EBVaGC, of which 15 reached statistical significance (*p* < 0.05) and three (PCDHB2, TMEM25, WWTR1) did not (*p* > 0.05) (Figure 1d). Because precursor miRNAs lack mature targeting activity, we focused subsequent analyses on the six mature EBV miRNAs and their nine associated downregulated host genes.

### 3.2. PPARα Expression Is Suppressed by EBV in Gastric Cancer Cells and Clinical Specimens

**Figure 2:**
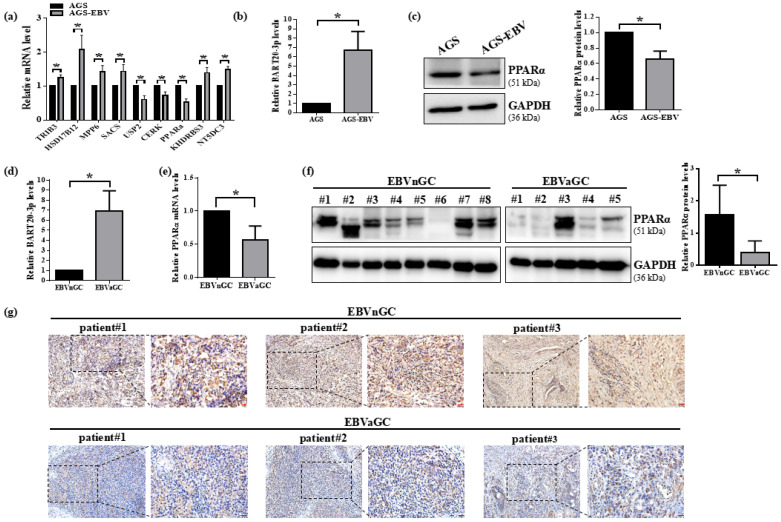
Expression of miR-BART20-3p and PPARα in EBV-infected gastric cancer cells and tissues. (**a**) qRT-PCR analysis of nine candidate DEGs in AGS-EBV versus AGS cells (*n* = 3; * *p* < 0.05 versus AGS), normalized to GAPDH. (**b**) miR-BART20-3p levels in AGS and AGS-EBV cells measured by qRT-PCR, normalized to U6, AGS set to 1 (*n* = 3; * *p* < 0.05). (**c**) Western blot of PPARα in AGS and AGS-EBV cells, with GAPDH as a loading control; relative quantification shown beneath (*n* = 3; * *p* < 0.05). (**d**) miR-BART20-3p expression in EBVnGC versus EBVaGC tissues (qRT-PCR, U6 normalization; *n* = 8; * *p* < 0.05). (**e**,**f**) PPARα mRNA (qRT-PCR, GAPDH normalization; *n* = 8) and protein (Western blot, GAPDH loading control; *n* = 5~8) in patient tissues. Accompanying bar graph quantifies relative expression levels of PPARα, normalized to GAPDH in tissue samples. Data are presented as mean ± SD. * *p* < 0.05. (**g**) Immunohistochemical staining of PPARα in EBVnGC and EBVaGC specimens. Scale bars: black, 50 μm; red, 20 μm.

To validate our bioinformatic predictions, we generated AGS-EBV cells by infecting parental AGS cells with recombinant EBV virions derived from Akata cells, confirming infection via EBER in situ hybridization (Figure S1). We then measured the mRNA levels of nine candidate DEGs (USP2, CERK, SACS, TRIB3, HSD17B12, MPP6, KHDRBS3, NT5DC3, and PPARα) in AGS cells with or without EBV infection (AGS-EBV). Despite being predicted as downregulated, six genes (TRIB3, HSD17B12, MPP6, SACS, KHDRBS3, NT5DC3) were paradoxically upregulated in AGS-EBV cells (Figure 2a), suggesting additional regulatory layers in vivo. In contrast, USP2, CERK, and PPARα mRNA were significantly reduced in AGS-EBV versus AGS cells, consistent with our dataset analyses. Because PPARα showed the greatest decrease and is a known target of miR-BART20-3p, we focused subsequent experiments on this pathway. We first confirmed that miR-BART20-3p is highly expressed in AGS-EBV cells compared with EBV–negative AGS controls (Figure 2b). Correspondingly, PPARα protein levels were markedly lower in AGS-EBV cells (Figure 2c), indicating an inverse relationship. To extend these findings to human tumors, we measured miR-BART20-3p and PPARα in EBV–negative (EBVnGC) and EBV-positive (EBVaGC) gastric cancer tissues. qRT-PCR showed elevated miR-BART20-3p in EBVaGC specimens (Figure 2d). Both qRT-PCR and Western blot analyses demonstrated significant reductions in PPARα mRNA and protein in EBVaGC versus EBVnGC samples (Figure 2e,f). Immunohistochemistry further confirmed markedly lower PPARα staining in EBVaGC tissues (Figure 2g). Together, these data indicate that EBV infection upregulates miR-BART20-3p and concomitantly suppresses PPARα expression in gastric cancer, supporting a functional miR-BART20-3p–PPARα axis in EBVaGC progression.

### 3.3. miR-BART20-3p Directly Binds the PPARα 3′-UTR to Suppress Its Expression

**Figure 3:**
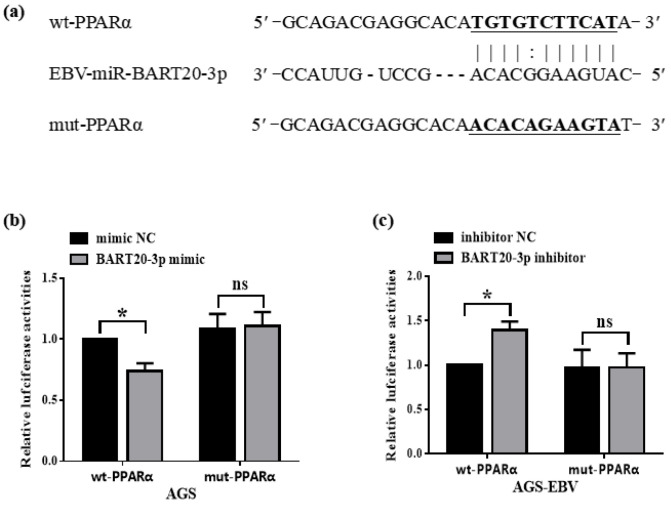
miR-BART20-3p directly targets the 3′-UTR of PPARα mRNA. (**a**) Predicted binding site of miR-BART20-3p within the wild-type PPARα 3′-UTR (wt-PPARα) with the seed-matching region underlined and bolded. The corresponding mutant PPARα 3′-UTR (mut-PPARα) includes nucleotide substitutions, also underlined and bolded. (**b**) Dual-luciferase reporter assay in AGS cells co-transfected with wt-PPARα or mut-PPARα reporter and either miR-BART20-3p mimic or mimic NC. Luciferase activity was measured 48 h after transfection and normalized to firefly luciferase. (**c**) Dual-luciferase reporter assay in AGS-EBV cells co-transfected with wt-PPARα or mut-PPARα reporter and either miR-BART20-3p inhibitor or inhibitor NC. Luciferase activity was measured 48 h after transfection and normalized to firefly luciferase. Data are presented as mean ± SD (*n* = 3). * *p* < 0.05; ns, not significant.

To determine whether miR-BART20-3p directly targets PPARα, we used RNAhybrid to predict a complementary seed-matching site within the PPARα 3′-UTR (Figure 3a). We then cloned either the wild-type 3′-UTR sequence (wt-PPARα) or a mutant bearing point mutations in the seed-binding region (mut-PPARα) downstream of a luciferase reporter. In AGS cells, co-transfection of wt-PPARα with miR-BART20-3p mimic led to a significant decrease in luciferase activity compared with mimic control, whereas the mut-PPARα reporter remained unchanged (Figure 3b). Conversely, in AGS-EBV cells, inhibition of miR-BART20-3p increased wt-PPARα reporter activity but had no effect on the mut-PPARα construct (Figure 3c). These results demonstrate that miR-BART20-3p binds specifically to the PPARα 3′-UTR to downregulate its expression.

### 3.4. miR-BART20-3p Enhances Gastric Cancer Cell Proliferation and Migration

**Figure 4:**
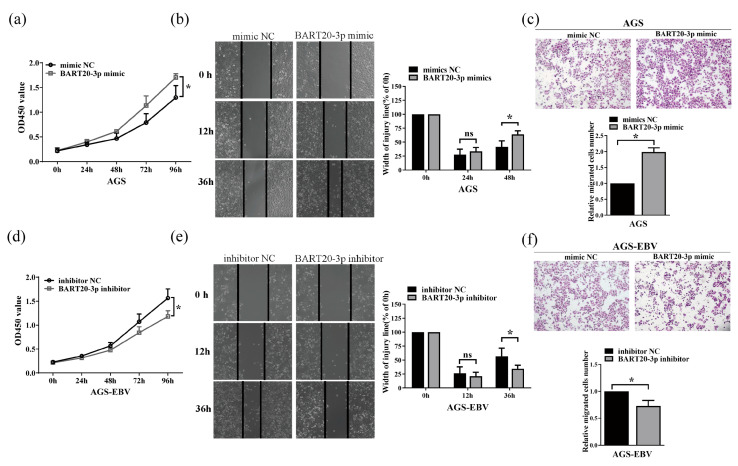
miR-BART20-3p promotes proliferation and migration of gastric cancer cells. AGS cells were transfected with miR-BART20-3p mimic or mimic negative control (mimic NC), and AGS-EBV cells with miR-BART20-3p inhibitor or inhibitor negative control (inhibitor NC). (**a**,**d**) CCK-8 assays measuring proliferation of AGS (**a**) and AGS-EBV (**d**) cells at 0, 24, 48, 72, and 96 h post-transfection (*n* = 3). (**b**,**e**) Wound-healing assays in AGS (**b**) and AGS-EBV (**e**) cells with representative images at 0 h, 12 h, and 36 h; wound closure quantified by ImageJ (*n* = 3). (**c**,**f**) Transwell migration assays in AGS (**c**) and AGS-EBV (**f**) cells; migrated cells counted and normalized to control (set as 1) (*n* = 3). Data are mean ± SD; * *p* < 0.05 versus respective NC; ns, not significant.

To assess the functional impact of miR-BART20-3p on gastric cancer cell behavior, we modulated its levels in AGS and AGS-EBV cells and measured proliferation and motility. Transfection of AGS cells with miR-BART20-3p mimic significantly increased cell proliferation over 48 h compared with mimic control, as determined by CCK-8 assays (Figure 4a). Conversely, inhibition of endogenous miR-BART20-3p in AGS-EBV cells reduced proliferation relative to inhibitor control (Figure 4d). We then evaluated cell migration using wound-healing and Transwell assays. AGS cells overexpressing miR-BART20-3p closed wounds more rapidly than controls, and migrated more efficiently through Transwell membranes (Figure 4b,c). In contrast, miR-BART20-3p inhibition in AGS-EBV cells slowed wound closure and decreased Transwell migration (Figure 4e,f). These data demonstrate that EBV-encoded miR-BART20-3p positively regulates both proliferation and migration in gastric cancer cells.

### 3.5. PPARα Knockdown Induces IL-6 Expression in Gastric Cancer Cells

**Figure 5:**
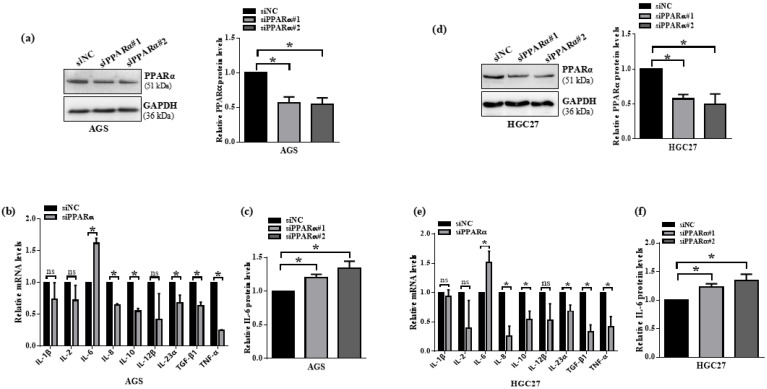
Downregulation of PPARα induces IL-6 expression in gastric cancer cells. AGS and HGC27 cells were transfected with two independent PPARα siRNAs (siPPARα#1, siPPARα#2) or scrambled control (siNC). (**a**,**d**) Western blot verification of PPARα knockdown in AGS (**a**) and HGC27 (**d**) cells. Densitometric quantification normalized to GAPDH and expressed relative to siNC (set to 1). (**b**,**e**) qRT-PCR analysis of cytokine mRNA levels—including IL-1β, IL-2, IL-6, IL-8, IL-10, IL-12β, IL-23α, TGF-β1, and TNF-α—in AGS (**b**) and HGC27 (**e**) cells 48 h post-transfection. Transcript levels were normalized to GAPDH and expressed relative to siNC (set to 1). Only IL-6 is significantly upregulated. (**c**,**f**) ELISA quantification of secreted IL-6 protein in culture supernatants of AGS (**c**) and HGC27 (**f**) cells following PPARα knockdown. Values are normalized to siNC (set to 1). Data represent mean ± SD of three independent experiments. * *p* < 0.05; ns, not significant.

Given PPARα’s established role in restraining pro-inflammatory cytokines such as IL-6, IL-1β, and TNF-α [19], and the importance of inflammation in gastric cancer progression [20], we next assessed whether loss of PPARα elevates inflammatory mediators in GC cells. Transfection of two independent PPARα siRNAs (siPPARα#1, siPPARα#2) into EBV-negative AGS and HGC27 cells achieved efficient knockdown at both mRNA and protein levels versus scrambled control (siNC) (Figure 5a,d). We then profiled a panel of cytokines (IL-1β, IL-2, IL-6, IL-8, IL-10, IL-12β, IL-23α, TGF-β1, TNF-α) by qRT-PCR. Only IL-6 mRNA was significantly upregulated upon PPARα silencing in both cell lines (Figure 5b,e). Consistently, ELISA measurements showed that IL-6 protein secretion increased following knockdown with either siPPARα construct (Figure 5c,f). These results demonstrate that PPARα depletion is sufficient to derepress IL-6 expression in gastric cancer cells, implicating the PPARα–IL-6 axis in tumor-associated inflammation.

### 3.6. miR-BART20-3p Suppresses PPARα and Induces IL-6 in EBV-Negative Gastric Cancer Cells

**Figure 6:**
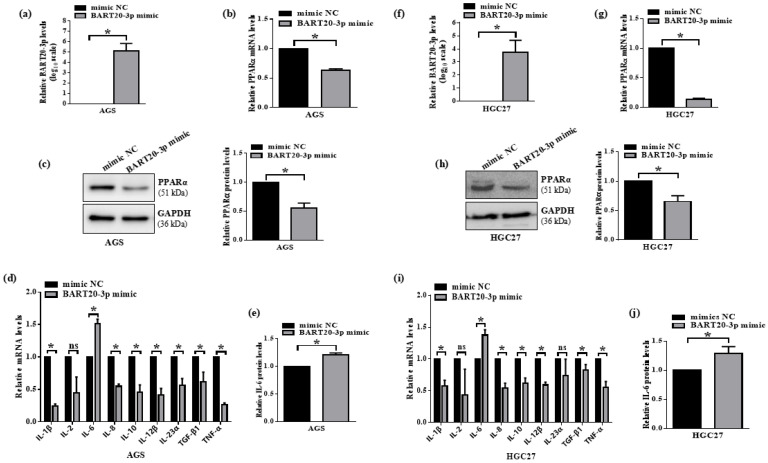
miR-BART20-3p suppresses PPARα and induces IL-6 in EBV-negative gastric cancer cells. AGS and HGC27 cells were transfected with miR-BART20-3p mimic or mimic negative control (mimic NC). (**a**,**f**) qRT-PCR quantification of miR-BART20-3p levels in AGS (**a**) and HGC27 (**f**) cells; U6 normalization, mimic NC set to 1. (**b**,**g**) qRT-PCR analysis of PPARα mRNA in AGS (**b**) and HGC27 (**g**) cells; GAPDH normalization, mimic NC set to 1. (**c**,**h**) Western blot and densitometric quantification of PPARα protein in AGS (**c**) and HGC27 (**h**) cells; normalized to GAPDH, mimic NC set to 1. (**d**,**i**) qRT-PCR profiling of nine inflammatory cytokine mRNAs (IL-1β, IL-2, IL-6, IL-8, IL-10, IL-12β, IL-23α, TGF-β1, TNF-α) in AGS (**d**) and HGC27 (**i**) cells; data normalized to GAPDH, mimic NC set to 1. (**e**,**j**) ELISA measurement of secreted IL-6 protein in AGS (**e**) and HGC27 (**j**) culture supernatants; values normalized to mimic NC (set to 1). Data represent mean ± SD of three independent experiments; * *p* < 0.05, ns = not significant.

To confirm that miR-BART20-3p downregulates PPARα and triggers IL-6 expression independently of EBV, we transfected miR-BART20-3p mimic or mimic NC into AGS and HGC27 cells. Successful miR-BART20-3p overexpression was verified by qRT-PCR, showing significant increases in AGS and HGC27 (Figure 6a,f). In AGS cells, miR-BART20-3p mimic reduced PPARα mRNA to 0.6 ± 0.05 and protein to 0.7 ± 0.08 relative to mimic NC (Figure 6b,c). HGC27 cells exhibited an even more pronounced reduction, with PPARα mRNA at 0.2 ± 0.03 and protein at 0.6 ± 0.09 (Figure 6g,h). USP2 and CERK were identified through the intersection of downregulated DEGs and ViRBase-predicted miR-BART20-3p targets, and were therefore evaluated by qRT-PCR. Both genes showed weaker and more variable regulation compared to PPARα (Figure S2). We next evaluated a panel of inflammatory cytokines by qRT-PCR. Only IL-6 mRNA increased significantly in both cell lines—to 1.6 ± 0.03 in AGS (Figure 6d) and 1.4 ± 0.11 in HGC27 (Figure 6i)—while other cytokines remained unchanged or decreased. ELISA confirmed corresponding elevations in secreted IL-6 protein: 1.2 ± 0.10–fold in AGS (Figure 6e) and 1.3 ± 0.14–fold in HGC27 (Figure 6j). Together, these data demonstrate that miR-BART20-3p directly inhibits PPARα, and thereby derepresses IL-6 expression in EBV-negative gastric cancer cells, mirroring the effects of PPARα knockdown.

### 3.7. miR-BART20-3p Inhibition Restores PPARα and Suppresses IL-6 in EBV-Positive Gastric Cancer Cells

**Figure 7:**
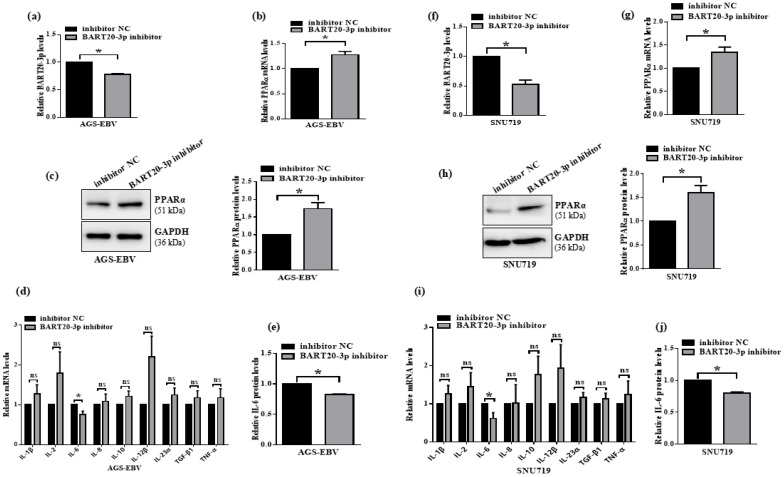
Inhibition of miR-BART20-3p restores PPARα and reduces IL-6 in EBV-positive gastric cancer cells. AGS-EBV and SNU719 cells were transfected with miR-BART20-3p inhibitor or inhibitor negative control (inhibitor NC). (**a**,**f**) qRT-PCR quantification of miR-BART20-3p levels in AGS-EBV (**a**) and SNU719 (**f**) cells; normalized to U6, inhibitor NC set to 1. (**b**,**g**) qRT-PCR measurement of PPARα mRNA in AGS-EBV (**b**) and SNU719 (**g**) cells; normalized to GAPDH, inhibitor NC set to 1. (**c**,**h**) Western blot and densitometric analysis of PPARα protein in AGS-EBV (**c**) and SNU719 (**h**) cells; normalized to GAPDH, inhibitor NC set to 1. (**d**,**i**) qRT-PCR profiling of nine inflammatory cytokine mRNAs (IL-1β, IL-2, IL-6, IL-8, IL-10, IL-12β, IL-23α, TGF-β1, TNF-α) in AGS-EBV (**d**) and SNU719 (**i**) cells; only IL-6 is significantly decreased. Data normalized to GAPDH, inhibitor NC set to 1. (**e**,**j**) ELISA quantification of secreted IL-6 protein in AGS-EBV (**e**) and SNU719 (**j**) culture supernatants; values normalized to inhibitor NC (set to 1). Data represent mean ± SD of three independent experiments; * *p* < 0.05, ns = not significant.

To determine whether blocking endogenous miR-BART20-3p reverses its effects on PPARα and IL-6, we transfected AGS-EBV and SNU719 cells with a miR-BART20-3p inhibitor (inhibitor NC as control). qRT-PCR confirmed that miR-BART20-3p levels fell by ~70% in AGS-EBV and ~60% in SNU719 cells (Figure 7a,f). In AGS-EBV cells, miR-BART20-3p inhibition increased PPARα mRNA to 1.3-fold and protein to 1.7-fold relative to control (Figure 7b,c). Similarly, in SNU719 cells, PPARα mRNA rose 1.4-fold and protein 1.6-fold following inhibitor treatment (Figure 7g,h). These data confirm that endogenous miR-BART20-3p suppresses PPARα expression in EBV-infected GC cells. We next profiled nine inflammation-related cytokines by qRT-PCR. Only IL-6 mRNA decreased significantly in both AGS-EBV and SNU719 cells after miR-BART20-3p inhibition (Figure 7d,i). ELISA assays corroborated these findings, showing reduced IL-6 secretion to ~0.8-fold in AGS-EBV and ~0.7-fold in SNU719 cells (Figure 7e,j). Together, these results demonstrate that antagonizing miR-BART20-3p upregulates PPARα and concomitantly downregulates IL-6, reinforcing the functional miR-BART20-3p–PPARα–IL-6 axis in EBV-associated gastric cancer.

### 3.8. miR-BART20-3p Regulates IL-6 Expression Through PPARα Modulation

**Figure 8:**
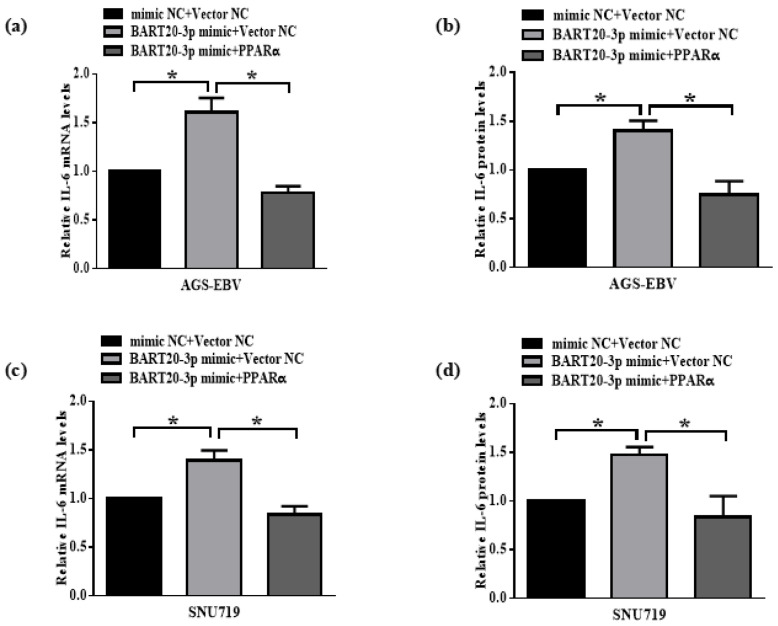
miR-BART20-3p induces IL-6 via PPARα in EBV-positive gastric cancer cells. AGS-EBV and SNU719 cells were first transfected with miR-BART20-3p mimic or mimic NC. After 12 h, cells received either a PPARα expression plasmid (pCDH-EF1-MCS-T2A-Puro-PPARα) or empty vector (pCDH-EF1-MCS-T2A-Puro) as Vector NC. Samples were collected 36 h after the second transfection. (**a**,**c**) qRT-PCR quantification of IL-6 mRNA in AGS-EBV (**a**) and SNU719 (**c**) cells; normalized to GAPDH, with mimic NC + vector set to 1. (**b**,**d**) ELISA measurement of secreted IL-6 protein in AGS-EBV (**b**) and SNU719 (**d**) cells; values normalized to mimic NC + vector (set to 1). Data are mean ± SD of three independent experiments; * *p* < 0.05 versus mimic NC + vector.

To confirm that miR-BART20-3p controls IL-6 by suppressing PPARα, we performed rescue experiments in EBV-infected GC cells. AGS-EBV cells were first transfected with miR-BART20-3p mimic or mimic NC. After 12 h, cells received either a PPARα expression vector or empty control. qRT-PCR showed that miR-BART20-3p mimic increased IL-6 mRNA to 1.8-fold over mimic NC, but co-expression of PPARα restored IL-6 levels to baseline (Figure 8a). Consistently, ELISA demonstrated that secreted IL-6 protein rose upon miR-BART20-3p overexpression and was normalized by PPARα reconstitution (Figure 8b). Similarly, in SNU719 cells treated with a miR-BART20-3p mimic or mimic NC, IL-6 mRNA and protein rose to ~1.4-fold; subsequent PPARα overexpression reversed this decrease, returning IL-6 to control levels (Figure 8c,d). These results establish that miR-BART20-3p induces IL-6 expression in EBV-positive gastric cancer cells by targeting PPARα, and that restoring PPARα is sufficient to counteract this effect.

## 4. Discussion

EBV is a ubiquitous γ-herpesvirus firmly established as an oncogenic driver in multiple cancers, including a distinct subclass of gastric cancer (EBVaGC). Although EBV-encoded microRNAs (miRNAs) have been detected in EBVaGC since their initial discovery in 2007 [21], the specific contributions of individual BART-region miRNAs to gastric tumorigenesis have remained incompletely defined. In particular, the role of miR-BART20-3p in modulating host gene networks and inflammation-driven tumor progression had not been fully explored.

In this study, we provide the first direct evidence that miR-BART20-3p is highly upregulated in EBVaGC tissues and EBV-infected gastric cell lines, and that it functions as a potent oncogenic signal. Overexpression of miR-BART20-3p in EBV–negative AGS cells markedly enhanced proliferation and migration, whereas inhibition of endogenous miR-BART20-3p in EBV-positive AGS-EBV cells produced the opposite effects. Through integrated bioinformatic analyses and dual-luciferase assays, we identified peroxisome proliferator-activated receptor α (PPARα) as a direct target of miR-BART20-3p. Manipulating PPARα levels—either by siRNA knockdown or by rescue with a PPARα expression vector—demonstrated that miR-BART20-3p regulates IL-6 expression in a PPARα-dependent manner. Specifically, loss of PPARα consistently led to selective upregulation of IL-6 mRNA and protein, whereas restoration of PPARα blunted the IL-6 increase triggered by miR-BART20-3p.

Our findings align with prior reports of other EBV BART miRNAs—such as miR-BART6-5p and miR-BART17-5p—that target tumor suppressors to promote glycolysis, proliferation, and invasion in EBVaGC [22,23]. Similar to miR-21’s ability to downregulate PPARα in atherosclerosis [24], miR-BART20-3p directly binds the PPARα 3′-UTR to inhibit its expression. PPARα is known to limit inflammation by repressing NF-κB and AP-1 signaling and downregulating IL-6, IL-1β, and TNF-α [19,25]. Consistent with fibrate-mediated PPARα activation reducing IL-6 in vivo [26,27], we show that PPARα depletion or miR-BART20-3p overexpression both elevate IL-6 in GC cells, linking viral miRNA activity to inflammation-driven tumor progression. Elevated IL-6 correlates with poor GC prognosis and activates JAK/STAT3 signaling to enhance proliferation, invasion, and lymphangiogenesis [28,29], underscoring the clinical relevance of our mechanistic insights.

This study has several limitations. Our functional assays were performed in vitro using two EBV-negative and two EBV-positive cell lines, and our tissue validation was limited to a modest number of primary specimens. These models may not capture the full genetic and microenvironmental heterogeneity of EBVaGC. It should also be acknowledged that both AGS-EBV and SNU719 cells express the full complement of EBV latent genes, including EBNA1, LMP1, LMP2A/B, as well as BART miRNAs, any of which could independently or cooperatively influence PPARα, IL-6, and related pathways. This inherent complexity implicates that while our data demonstrate a critical role for miR-BART20-3p, we cannot exclude additional contributions from other viral factors. We focused predominantly on IL-6 as the downstream inflammatory mediator, but other cytokines and chemokines regulated by PPARα or additional BART miRNAs may also contribute. Moreover, although our rescue experiments confirm a PPARα-dependent mechanism, we have not yet delineated the precise downstream signaling events—such as NF-κB or STAT3 activation—in response to IL-6 in this context.

Future work should extend these findings in vivo by employing xenograft or genetically engineered mouse models of EBVaGC to evaluate the impact of miR-BART20-3p inhibition or PPARα activation on tumor growth and metastasis. Tumor development and progression depend not only on viral factors but also on the inflammatory microenvironment, host genetics, and co-infecting microbes. To validate the specificity and reversibility of the miR-BART20-3p–PPARα–IL-6 axis, future studies should include CRISPR/Cas9 knockout of key EBV genes in naturally infected cell lines (e.g., SNU719) to dissect the contribution of individual viral components. Comprehensive, multivariate clinical analyses that incorporate patient age, sex, *Helicobacter pylori* status, and other relevant metadata will be essential for distinguishing EBV-specific regulation from broader gastric cancer drivers. Finally, advanced models such as gastric organoids and epithelial–immune co-culture systems will clarify whether epithelial EBV infection alone is sufficient to trigger this regulatory circuit. Comprehensive profiling of cytokine networks and transcription factor activity will clarify how IL-6 engages pro-tumorigenic pathways downstream of PPARα suppression. Longitudinal studies in patient cohorts could assess whether circulating levels of miR-BART20-3p or IL-6 predict disease progression or response to therapy. Finally, combining PPARα agonists (e.g., fibrates) with inhibitor of JAK/STAT3 or NF-κB may offer synergistic therapeutic strategies tailored to EBVaGC. Given that EBV is associated with several other malignancies including nasopharyngeal carcinoma and lymphoma, future studies are needed to determine whether the miR-BART20-3p–PPARα regulatory axis operates similarly in other EBV-associated cancers.

In summary, our work uncovers a novel oncogenic axis in EBV-associated gastric cancer, whereby EBV-encoded miR-BART20-3p directly targets PPARα to unleash IL-6–driven inflammatory signaling, thereby promoting tumor cell proliferation and migration. These findings identify EBV-encoded miR-BART20-3p as one of major modulators of the PPARα–IL-6 axis in EBVaGC. Targeting this miR-BART20-3p–PPARα–IL-6 pathway holds promise both as a biomarker of disease activity and as a foundation for therapeutic intervention in EBVaGC.

## Data Availability

All data are available from the corresponding author on reasonable request.

## Supplementary Materials

The following supporting information can be downloaded at: https://www.mdpi.com/article/10.3390/microorganisms13071514/s1, Figure S1: Establishment and validation of EBV-infected gastric cancer cell lines. Figure S2: miR-BART20-3p regulation of USP2 and CERK mRNA levels determined by qRT-PCR. Table S1: Primer sequences used for qRT-PCR analysis. Table S2: Oligonucleotide sequences of miR-BART20-3p and siRNAs targeting PPARα.
