# Supplementary material for: EBV-Derived miR-BART20-3p Influences Proliferation and Migration in EBV-Positive Gastric Cancer Models by Suppressing PPARα
Source: Microorganisms. 2025 Jun 28;13(7):1514. doi: 10.3390/microorganisms13071514 (PMC12298216; doi:10.3390/microorganisms13071514)
Supplement: Supplementary file 1 [file microorganisms-13-01514-s001.zip › microorganisms-3694643-supplementary.pdf]

Table S1. Oligonucleotide sequences of miR-BART20-3p and siRNAs targeting PPARα

Genes	Sequences
mimic NC	5'-UUCUCCGAACGUGUCACGUTT-3'
	5'-ACGUGACACGUUCGGAGAATT-3
BART20-3p mimic	5'-CAUGAAGGCACAGCCUGUUACC-3'
	5'-UAACAGGCUGUGCCUUCAUGUU-3'
inhibitor NC	5'-CAGUACUUUUGUGUAGUACAA-3'
BART20-3p inhibitor	5'-GGUAACAGGCUGUGCCUUCAUG-3'
siNC	5'-GCAGGAGGGUAUUGUACAUTT-3'
	5'-AUGUACAAUACCCUCCUGCTT-3'
siPPARα#1	5'-GAUCUAGAGAGCCCGUUAUTT-3'
	5'-AUAACGGGCUCUCUAGAUCTT-3'
siPPARα#2	5'-GCAGGAGGGUAUUGUACAUTT-3'
	5'-AUGUACAAUACCCUCCUGCTT-3'

Table S2. Primer sequences used for qRT-PCR analysis

Genes	Primer sequences
USP2	F: 5'-AAAGGCCGACAGATGTGGAG-3'<br>R: 5'-AGGGGTCGAAGACCGTAGAA3'
CERK	F: 5'-TATCAACCCGTTTGGAGGAAAAG-3'<br>R: 5'-ATGGAGGCTAAGGTGAACAGT-3'
SACS	F: 5'-CCAAGGTCCCTTTTCCCAAGT-3'<br>R: 5'-GTACATGGTCTTCCTCGGGT-3'
TRIB3	F: 5'-CGCTGACCGTGAGAGGAAGAAG-3'<br>R: 5'-TTGTCCCACAGGGAATCATCTG-3'
HSD17B12	F: 5'-TAGTGGAAAGCCATGGAGAGC-3'<br>R: 5'-AGTACTACCTGTGACAACTGCC-3'
MPP6	F: 5'-TGTCAACCCACAAGCACTGA-3'<br>R: 5'-CCTTGTGCATGGCACGTAAC-3'
KHDRBS3	F: 5'-AACAGCTATAGCACCCCAGC-3'<br>R: 5'-CCACTCTTCTTGCCCGTAGG-3'
NT5DC3	F: 5'-CATGAAGCGCTACCTGTGGG-3'<br>R: 5'-AATGGAAGGAACCAATTCTTCTGTG-3'
PPARα	F: 5'-GCGAACGATTCGACTCAAGC-3'<br>R: 5'-CATCCCGACAGAAAGGCACT-3'
IL-1β	F: 5'-TTCGAGGCACAAGGCACAA-3'<br>R: 5'-TGGCTGCTTCAGACACTTGAG-3'
IL-2	F: 5'-GAATCCCAAACTCACCAGGA-3'<br>R: 5'-GATGTTTCAGTTCTGTGGCCTTC-3'
IL-6	F: 5'-AGTTCCTGCAGAAAAAGGCAAAGAA-3'<br>R: 5'-GCGCAGAATGAGATGAGTTGT-3'
IL-8	F: 5'-ACCGGAAGGAACCATCTCAC-3'<br>R: 5'-GGCAAAACTGCACCTTCACAC-3'
IL-12β	F: 5'-AGGGGACAACAAGGAGTATGAG-3'<br>R: 5'-AGGGAGAAGTAGGAATGTGGAG-3'
IL-23α	F: 5'-TTCTGCTTGCAAAGGATCCA-3'<br>R: 5'-AATATCCGATCCTAGCAGCTTCTC-3'
TGF-β1	F: 5'-GCACGTGGAGCTGTACCA-3'<br>R: 5'-AAGATAACCACTCTGGCGAGTC-3'
TNF-α	F: 5'-TGCACTTTGGAGTGATCGGC-3'<br>R: 5'-GCTTGAGGGTTTGCTACAACA-3'
GAPDH	F: 5'-ATGGGGAAGGTGAAGGTCG-3'<br>R: 5'-GGGGTCATTGATGGCAACAATA-3'
BART20-3p	F: 5'-CGCATGAAGGCACAGCCT-3'<br>R: 5'-AGTGCAGGGTCCGAGGTATT-3'
miR-U6	F: 5'-CGCGACGCAAATTCGTGA-3'<br>R: 5'-AGTGCAGGGTCCGAGGTATT-3'

EBV-infected AGS cells were established

To further investigate the differential expression of the 9 downregulated DEGs associated with EBV miRNAs in EBVaGC cell lines. Akata cells, which secrete recombinant EBV virions carrying green fluorescent protein (GFP) and confer puromycin resistance, were co-cultured with AGS cells to facilitate EBV infection via the cell-to-cell transmission method. Following infection, AGS cells were continuously selected using G418 to establish EBV-infected AGS cell lines, designated as AGS-EBV cells. Green fluorescence of recombinant EBV virions was observed in AGS-EBV cells but not in AGS cells (Figure S1a). Subsequent in situ hybridization confirmed abundant EBER expression in most cells within the EBV-infected cultures (Figure S1a), and the western blot analysis verified the presence of EBNA1 protein in AGS-EBV cells (Figure S1b). Collectively, these results demonstrate AGS-EBV cells were established by co-culturing AGS cells with Akata cells, which are capable of secreting recombinant Epstein-Barr virus.

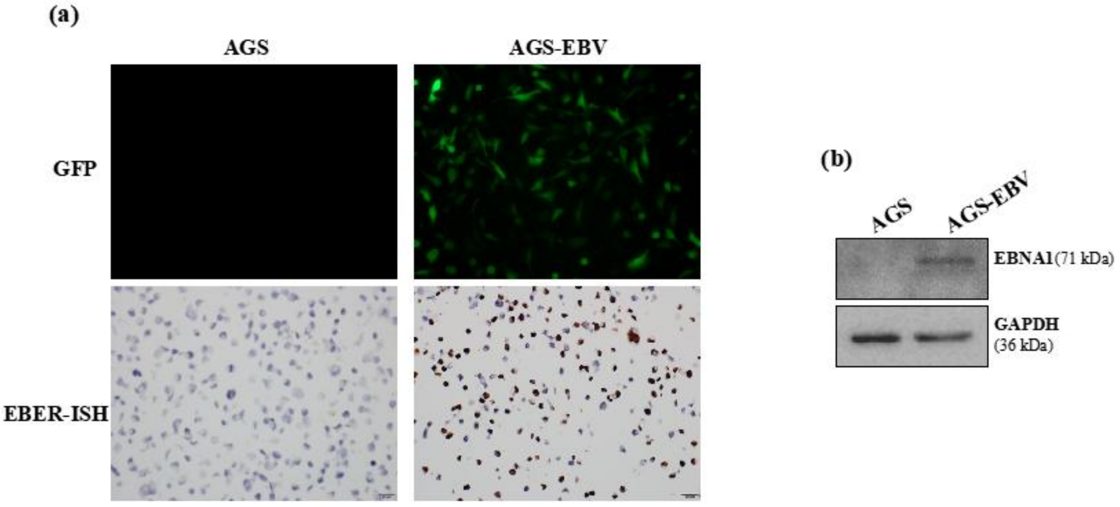

Figure S1. Establishment and validation of EBV-infected gastric cancer cell lines. (**a**) The microscopic analysis was performed to examine fluorescence in AGS and AGS-EBV cells (upper), while in situ hybridization staining was used to analyze EBER staining in EBV-infected cells. Scale bars=20 μm (lower). (**b**) Western blot analysis of EBNA1 protein levels in AGS-EBV cells, with AGS cells serving as the control. GAPDH serving as the internal reference gene (n=3; $*p < 0.05$).

miR-BART20-3p regulation of USP2 and CERK mRNA levels determined by qRT-PCR

To determine whether PPARα was uniquely regulated by miR-BART20-3p, we evaluated two additional candidates—USP2 and CERK—identified through the intersection of downregulated DEGs and ViRBase-predicted miR-BART20-3p targets, by qRT-PCR. AGS cells were transfected with miR-BART20-3p mimic, and AGS-EBV cells with miR-BART20-3p inhibitor. In AGS cells, mimic transfection reduced USP2 mRNA to 0.80 ± 0.04 relative to mimic NC (Figure S2a), whereas USP2 levels in AGS-EBV cells did not significantly change with inhibitor treatment compared to inhibitor NC (Figure S2b). CERK mRNA expression remained unaffected by miR-BART20-3p modulation in both cell lines. These data, alongside fold-change comparisons in EBV-negative and EBV-positive gastric cancer cells, reinforce PPARα as the most consistent and functionally relevant direct target of miR-BART20-3p in EBV-associated gastric cancer.

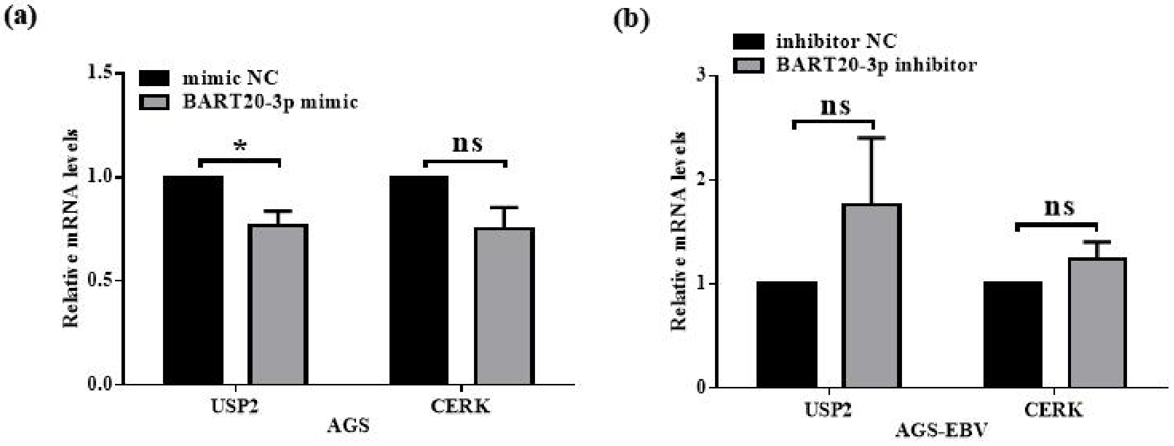

Figure S2. miR-BART20-3p regulation of USP2 and CERK mRNA levels determined by qRT-PCR. **(a)** USP2 and CERK mRNA in AGS cells transfected with miR-BART20-3p mimic or mimic NC; normalized to GAPDH, mimic NC set to 1 (n = 3; **p* < 0.05, ns = not significant). **(b)** USP2 and CERK mRNA in AGS-EBV cells transfected with miR-BART20-3p inhibitor or inhibitor NC; normalized to GAPDH, inhibitor NC set to 1 (n = 3; ns = not significant).
